# Transcutaneous electrical acupoint stimulation for reducing cognitive dysfunction in lumbar spine surgery: A randomized, controlled trail

**DOI:** 10.3389/fnagi.2022.1034998

**Published:** 2022-12-05

**Authors:** Li-feng Wang, Wei-dong Liang, Bing-yu Wang, Ming-ling Guo, Jian-shun Zhou, Li Chen, Mao-lin Zhong, Jun-ming Ye

**Affiliations:** ^1^Department of Anesthesiology, Suzhou Medical College of Soochow University, Suzhou, Jiangsu, China; ^2^Department of Anesthesiology, First Affiliated Hospital of Gannan Medical College, Ganzhou, Jiangxi, China; ^3^Department of Anesthesiology, Gannan Medical College, Ganzhou, Jiangxi, China

**Keywords:** POCD, TEAS, aging, lumbar spine surgery, neuroinflammation

## Abstract

**Objective:**

This study aimed to evaluate the effect of perioperative transcutaneous electrical acupoint stimulation (TEAS) on postoperative cognitive dysfunction (POCD) in older patients with lumbar spine surgery.

**Methods:**

Older patients (aged 60–80 years old) receiving lumbar spine surgery under general anesthesia were randomly divided into group A, 3-day intervention group; group B, 7-day intervention group; control group C, sham TEAS group, selected “Baihui” (GV 20) and “Dazhui” (GV 14) point was intervened once 30 min before operation with “HANS” transcutaneous electrical stimulation device, and then once a day after operation for 30 min each time. The primary outcome was the incidence of postoperative cognitive impairment assessed by the use of the Mini Mental Rating Scale (MMSE), patients developed POCD according to the Z score method. The secondary outcome was serum interleukin-6 (IL-6), tumor Necrosis factor α (TNF-α), neuron-specific enolase (NSE), and S100β protein levels.

**Results:**

Three days after surgery, the incidence of POCD in groups A((22.4%)) and B ((18.3%)) were lower than those in group C ((42.9%)) (*P* < 0.05). There was no significant difference between groups A and B (*P* > 0.05). Seven days after surgery, the incidence of POCD in group B (18.3%) was lower than that in groups A (26.5%) and B (42.9%), and the comparison between groups B and C was statistically significant (*P* < 0.05). On the 3rd and 7th days after surgery, the levels of IL-6, TNF-α, NSE, and S100β in the two TEAS groups were lower than those in the sham TEAS group (*P* < 0.01), but higher than the preoperative levels in the three groups (*P* < 0.01).

**Conclusion:**

It seems that Perioperative TEAS intervention could reduce the level of inflammatory factors IL-6, TNF-α in the blood of older patients with lumbar spine surgery, and reduce the incidence of POCD.

**Clinical trial registration:**

www.chictr.org.cn, identifier ChiCTR2200063030.

## Introduction

POCD (postoperative cognitive dysfunction, POCD) is a complication characterized by impaired memory, decreased information processing ability and attention, such as changes in mental mood and personality (Le et al., 2014), which seriously reduces the quality of life and negatively affects social stability (Zarbo et al., 2018). POCD is more common in older patients, and the risk of developing POCD 7 days after surgery is approximately 25–40% (Wei et al., 2019), which greatly increases the 1-year postoperative mortality in older patients (Leslie, 2017). At present, prevention and treatment methods for POCD are very limited. Therefore, effective intervention methods are urgently needed.

Neuroinflammation is hypothesized to be an important contributor to the pathogenesis of POCD and its involvement is considered to be the most credible theory (Wang et al., 2013; Lin et al., 2021; Suo and Wang, 2021). In particular, inflammation in the hippocampus may play a key role in the pathogenesis of POCD. Stimulated by surgical trauma, the peripheral immune system activates and releases inflammatory factors, leading to neuroinflammation through disruption of the blood-brain barrier (BBB), direct neural pathways, or transport to the brain (Kline et al., 2016; Yang et al., 2017; Femenía et al., 2018). Studies show that the inflammatory factors interleukin-6 (IL-6) and tumor necrosis factor α (TNF-α) in the peripheral blood are closely related to the pathogenesis of POCD (Lei et al., 2020; Yang et al., 2022).

Acupoint electrical stimulation is an important part of traditional Chinese medicine in China. Acupoint electrical stimulation has been used for many years in clinical practice and researchers have accumulated rich experience, which has been applied to treatment for many learning and memory impairment diseases, such as dementia (Cai et al., 2019; Xie et al., 2021) and cerebral apoplexy (Zhao et al., 2018). Compared with drug therapy, acupoint electrical stimulation has the unique advantages of safety and fewer side effects. Transcutaneous electrical nerve stimulation (TENS) was developed according to the theory of pain gate control. TENS can input a specific low-frequency pulse current into the human body through the skin to relieve pain and treat diseases. In recent years, people have applied TENS to acupuncture points in traditional medicine to create transcutaneous electrical acupoint stimulation (TEAS), which has the dual advantages of electrical stimulation and acupuncture. Because of its simple operation, non-invasiveness, painlessness and other characteristics, TEAS is widely used in clinical practice. Studies show that transcutaneous electrical stimulation can be used to reduce the dose of opioids (Yin et al., 2018), reduce the occurrence of nausea and vomiting (Chen et al., 2020), and promote the recovery of gastrointestinal function (Li et al., 2021). The “Baihui” (GV 20) and “Dazhui” (GV 14) acupoints belong to the governor vessel, and the governor vessel enters the collateral brain. Electroacupuncture at “Baihui” and “Dazhui” can reduce the central inflammatory response and improve cognitive impairment caused by mental diseases such as anxiety and depression (Park et al., 2019; Liu et al., 2020; Chen et al., 2021). However, there are few clinical studies on the use of transcutaneous electrical stimulation of these two acupoints for reducing POCD. The purpose of this study was to investigate whether transcutaneous electrical stimulation of “Baihui” and “Dazhui” can reduce POCD and to observe the effects of different treatment durations.

## Materials and methods

We conducted this prospective, randomized, double-blind, intervention-controlled clinical trial in the First Affiliated Hospital of Gannan Medical College of Jiangxi Province. This protocol was approved by the Ethics Committee of the First Affiliated Hospital of Gannan Medical College (LLSC-2020070203) in accordance with the Declaration of Helsinki. The project has been registered in the China Clinical Trial Registration Center (registration number: ChiCTR2200063030).

### Participants

All patients signed informed consent before this study. The selection criteria for patients were as follows: age 60–80 years, receiving lumbar spine surgery, ASA I-III, can speak Mandarin and can communicate, an education level > 9 years, a mini-mental state examination (MMSE) score ≥ 26, no obvious abnormalities in heart, lung, liver or kidney function, and operation time less than 3 h.

Exclusion criteria included (1) patients with preoperative cognitive impairment, dementia and delirium history; (2) patients with major depression, schizophrenia, and other psychiatric and neurological diseases or who had taken antipsychotics or antidepressants; (3) patients using anti-inflammatory drugs, glucocorticoids or other hormonal drugs, or with alcohol or drug dependence; (4) patients with severe heart, lung, liver or kidney dysfunction; (5) patients with hearing or visual impairment inhibiting their ability to complete the test; (6) patients with a skin ulceration or infection around the target acupoints.

Drop out criteria included (1) The patient or the client request to withdraw from the study during the observation period; (2) The patient or the client are unwilling to cooperate after surgery; (3) patients with intraoperative bleeding > 800 ml and hospitalized for 3 months or more.

### Sample size calculation

The sample size for this study was calculated using Med Cal 15.2.2 software. A previous study reported that the incidence of POCD is 25–40% (Moller et al., 1998; Rasmussen et al., 1999). According to our preliminary experiment, the incidence of POCD decreased from 40 to 20% after 7 days TEAS, with the statistical power of 90% and a two-sided significance level of 0.05. Considering a loss to follow-up rate of 10–20%, 52 patients in each group were required to detect a statistical significance and a total of 156 subjects were enrolled in our study. Patients were assigned to the TEAS 3-day group (group A), TEAS 7-day group (group B), or sham TEAS group (group C).

### Randomization and blindness

Using SPSS23.0 software, click the Random Number generator of Transform, first set the fixed random number seed 20220807, and generated random numbers for each case through Rv. Uniform random function. Then the SPSS Visual Binning function was used to group the samples into three groups with the same sample size in order of random number.

Group concealment: Put each grouping scheme into an opaque envelope, wrote a code on the outside of the envelope, sealed it, and handed it over to the researcher. After the subjects entered the study, the subjects were numbered one by one, and then the envelopes with corresponding numbers were opened and grouped according to the distribution scheme in the envelopes. Patients were assigned to the TEAS 3-day group, TEAS 7-day group, or sham TEAS group according to a 1:1:1 random number. Patients knew the number but did not know how to group, and the randomization process was completed by a study administrator not involved in the trial.

### Anesthesia management

All patients underwent tracheal intubation under general anesthesia without preoperative medication. After entering the operating room, intravenous access was established, and a multifunctional monitor was connected to monitor hemodynamics, electrocardiography (ECG), non-invasive blood pressure (BP), blood oxygen saturation (SPO_2_), and heart rate (HR). Anesthesia was induced using the following drugs: midazolam 0.04–0.06 mg/kg, sufentanil 0.5–1 μg/kg, and rocuronium 0.4–0.6 mg/kg. Mechanical ventilation was performed after tracheal intubation, and the adjustment parameters were tidal volume 8–10 ml/kg, respiratory rate 12–16 breaths/min, expiratory inhalation ratio 1:2, and oxygen flow rate 2 L/min to maintain end-tidal carbon dioxide partial pressure (PECO_2_) at 35–45 mmHg. After induction, left radial artery puncture and manometry were performed to facilitate monitoring of arterial blood pressure and blood gas analysis.

### Anesthesia maintenance

During the operation, muscle relaxants, including remifentanil 0.25–0.4 μg/(kg. min), dexmedetomidine 0.2–0.4 μg/(kg. h), and propofol 6–9 mg/(kg. h) were administered intermittently, and the bispectral index (bis) was maintained at 40–60.

### Postoperative analgesia

All patients received postoperative patient-controlled intravenous analgesia (PCIA). PCIA configuration: Sufentanil 5–10 μg/kg + 0.9% sodium chloride to make 100 ml.

### Intervention

**FIGURE 1:**
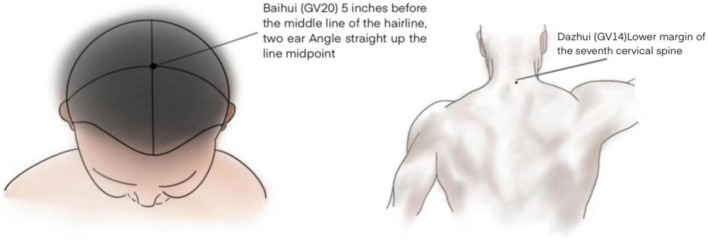
Location of acupoints.

The patient’s “Baihui” (the intersection of the midline of the top of the head of GV20 and the line connecting the two ear tips) and “Dazhui” (in the depression under the spinous process of the seventh cervical vertebra of GV14) were selected as the acupoints for intervention, and two trained researchers performed perioperative intervention (Figure 1). Intervention time: group A received TEAS 30 min before the surgery and up to 3 days after the surgery, group B received TEAS 30 min before the surgery and up to 7 days after the surgery, and group C received sham TEAS 30 min before the surgery and up to 7 days after the operation for 30 min, once a day. In the TEAS group, a transcutaneous acupoint electrical stimulator (HANS-200A, Nanjing Jisheng Medical Technology Co., Ltd., Suzhou, China) was used to create sparse and dense waves with a frequency of 2/100 Hz and an intensity of less than 10 mA. When the stimulation intensity is too high, the patient feels uncomfortable, so the intensity is reduced until it is comfortable. The sham TEAS group used the placebo-type Han’s acupoint nerve stimulator (sham HANS). It causes a feeling of numbness in the acupoints but has no therapeutic effect. The comforting effect of this device has been confirmed in research (Lambert et al., 2011).

### Outcomes

#### Primary outcome

The primary outcome of this study was changes in the MMSE scores at 1 day before surgery and at 3 and 7 days after surgery, and the incidence of POCD was calculated based on the “Z” score. The evaluators for this study received specialized training in neuropsychological assessments. The MMSE assessment scale was used for evaluation. The MMSE is a 30-point questionnaire for orientation (time and place), memory (immediate and short-term), attention, computation, and language (naming, repetition, pronunciation, reading and writing). In this study, some healthy people aged 60–80 years were selected from the anesthesia clinic or accompanying family members. The exclusion criteria were the same as the selection criteria. These older adults had not undergone surgery in the past 6 months, and had not planned to undergo surgery in the next 3 months. No surgery was planned for the month. The same neuropsychological test administered to the healthy control group was repeated twice at each time point, and the average value was taken as the learning effect. The subjects were also evaluated twice, the average value was taken, the difference was calculated, and the learning effect M was subtracted. For its actual difference, a Z score was calculated. Calculation formula: [(postoperative MMSE-preoperative MMSE)-ΔX MMSE standard population]/[SD (ΔX MMSE standard population)]. The ΔX MMSE standard population refers to the mean MMSE change in the normal population, and the standard deviation (ΔX MMSE standard population) refers to the standard deviation of the MMSE change in the normal population measured in a cognitively normal sample. In this study, ΔX MMSE standard population = 1.4, SD (ΔX MMSE standard population) = 0.5 was used to calculate Z score, Z score ≥ 1.96 was determined to be abnormal, and POCD was diagnosed. Once the patient developed POCD, 5 mg of haloperidol was injected intramuscularly.

#### Secondary outcomes

Basic patient data were recorded, including sex, age, body mass index, ASA classification, history of underlying diseases (hypertension, diabetes, coronary heart disease), and smoking history.

Preoperative and intraoperative data included operation time, anesthesia time, intraoperative blood loss, infusion volume, recovery time, extubation time, and postoperative VAS score.

Venous blood was drawn from patients at different time points to measure related indicators: before TEAS treatment and 3 and 7 days after TEAS treatment, 3 ml of venous blood was drawn to measure IL-6, TNF-α, S100β, and NSE.

#### Enzyme-linked immunosorbent assay

Blood samples were collected and centrifuged at 3,000 × g for calibration, and the supernatant was taken and stored at –80°C until the samples were centrally processed and analyzed. Repeat quantification was performed with a commercially available enzyme-linked immunosorbent assay (ELISA) kit (Proteintech, USA) following the manufacturer’s protocol. Average values were taken for analysis. Batch determination of all clinical data was performed by the same laboratory technician every 3 months.

#### Statistical analyses

SPSS 23.0 statistical software was used for statistical analysis. The mean ± SD was used for measurement data conforming to a normal distribution, ANOVA was used for intergroup comparisons, M (P25, P75) was used for measurement data conforming to a normal distribution, and the Kruskal-Wallis test was used for intergroup comparisons. The χ^2^-test was used for counting data. The time and group differences in serologic indexes (IL-6, TNF-α, S100B, and NSE), MMSE score and VAS score were analyzed by repeated-measures ANOVA. A *P*-value < 0.05 was considered statistically significant.

## Results

### Clinical characteristics of patients and surgical results

**FIGURE 2:**
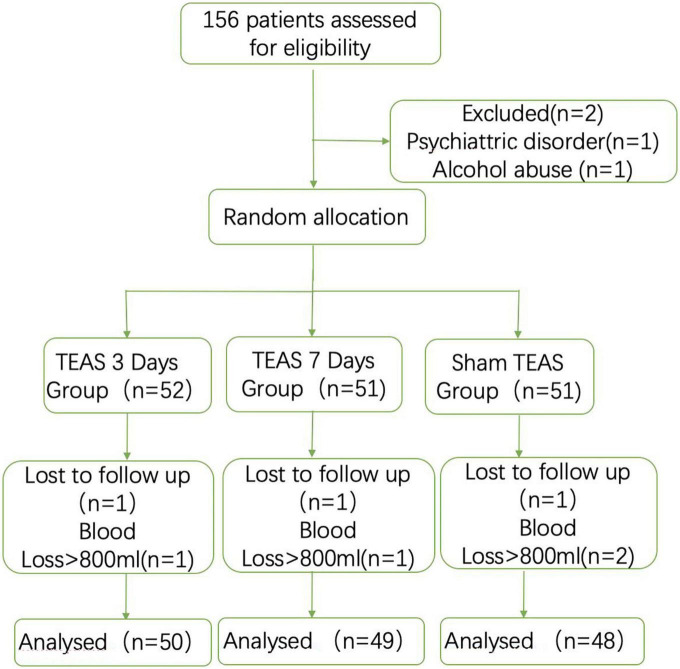
Flow diagram of study.

A total of 156 patients were planned to participate in this trial but some were unwilling to cooperate after surgery due to a history of neurological and psychiatric disorders, alcohol abuse [*n* = 2 (1.2%)], intraoperative blood loss ≥ 800 mL [*n* = 4 (2.5%)], and treatment [*n* = 3 (1.9%)]. These 9 patients were dropped out from the study, leaving 147 patients for the analysis (Figure 2). The patients included in the final study were all Han Chinese, and the general characteristics of the patients included sex [female, 87 (59.1%)], age (69.1 ± 5.55 years), BMI (23.3 ± 1.84 kg/m2), American Society of Anesthesiologists (ASA II) [90 (61.2%)], smokers [39 (26.5%)], history of diabetes [55 (37.4%)], hypertension [118 (80.2%)], and coronary heart disease [48 (32.6%)] (Table 1). There was no significant difference in operation time, anesthesia time, recovery time, extubation time, intraoperative infusion volume, blood loss or postoperative VAS score among the three groups. (*P* > 0.05) (Table 1).

**TABLE 1:** General information of three groups of patients and intraoperative and postoperative variable.

Characteristics	TEAS 3 days group	TEAS 7 days group	Sham TEAS group	*P*-value
Age (year)	65.0 (61.75–71.0)	66 (63.0–70.5)	66 (63.25–69.0)	0.658
Sex (man/woman), *n*	25/26	31/18	31/17	0.766
BMI (kg/m^2^)	23.44 ± 0.28	23.47 ± 0.26	22.99 ± 0.25	0.355
ASA (l/II/III)	13/26/12	13/25/11	13/27/8	0.631
Hypertension [*n* (%)]	38 (75)	36 (73)	35 (73)	0.121
Diabetes mellitus [*n* (%)]	18 (35)	16 (33)	18 (38)	0.928
Coronary heart disease [*n* (%)]	15 (29)	17 (35)	17 (35)	0.479
Hypoproteinemia [*n* (%)]	8 (16)	9 (18)	10 (21)	0.826
Smoker [*n* (%)]	13 (25)	12 (24)	12 (25)	0.919
Fluid infusion volume(ml)	1350.00 (1287.50–1500.00)	1400.00 (1300.00–1500.00)	1400.00 (1300.00–1587.50)	0.257
Volume of blood loss(ml)	350.00 (310.00–402.50)	370.00 (320.00–450.00)	385.00 (342.50–445.00)	0.29
Operation time (min)	142.82 ± 2.10	138.04 ± 1.87	140.65 ± 2.08	0.246
Anesthesia time (min)	145.56 ± 2.07	142.29 ± 1.71	1444.33 ± 2.01	0.482
Awakening time(min)	42.00 (38.00–48.50)	43.00 (38.00–52.50)	54.00 (41.25–53.75)	0.37
Extubation time(min)	50.00 (45.75–55.00)	50.00 (46.00–59.00)	52.00 (50.00–59.75)	0.31
VAS scores (3 days and 7 days)	4.90 ± 1.00 2.34 ± 0.63	5.49 ± 1.24 2.27 ± 0.64	5.04 ± 1.07 5.04 ± 1.07	0.052

Data are presented as a number or means ± SD or median and range or numbers and percentages.

BMI, body mass index; ASA, American Society of Anesthesiologists.

### Mini mental rating scale and incidence of postoperative cognitive dysfunction:

**FIGURE 3:**
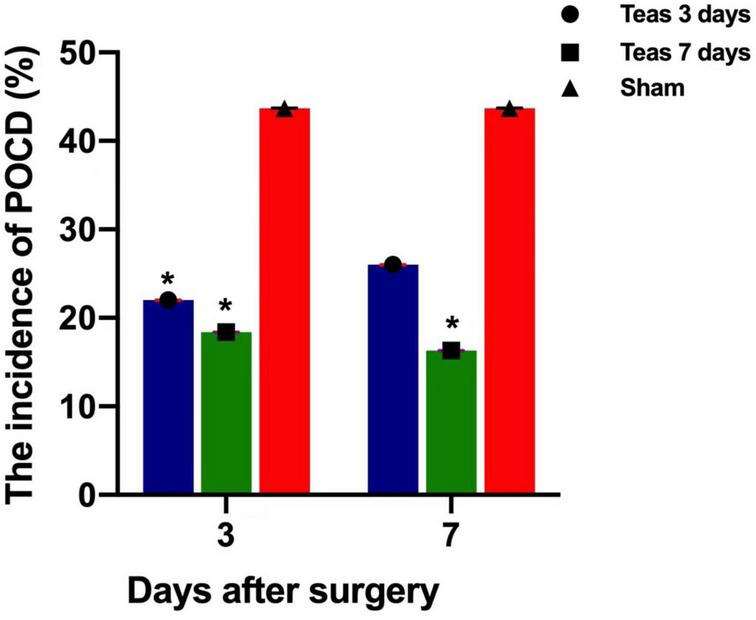
Postoperative incidence of POCD in three groups (%).*P < 0.05 vs. group C; *P < 0.05 vs. group C.

The multivariate analysis of variance shown that there was no significant difference in MMSE scores between the three groups. However, the incidence of POCD measured by Z score was different. On the 3rd day after the operation, POCD occurred in 11 (22%), 9 (18.4%) and 21 (43.7%) patients in groups A, B, and C, respectively, and there was no significant difference between groups A and B (*P* = 0.616). The two groups were significantly different from group C (*P* = 0.031, *P* = 0.015). On the 7th day after the operation, there was one new case in group A, a total of 13 cases (26%), and no new cases in group B and group C. POCD still occurred in 8 patients (16.3%) and 21 patients (43.7%). There was no difference between group A and groups B and C (*P* = 0.218, *P* = 0.09), but there was a difference between groups B and C (*P* = 0.004) (Tables 2, 3 and Figure 3).

**TABLE 2:** MMSE scores in three groups.

Group	MMSE (preoperative)	MMSE (TEAS 3 days)	MMSE (TEAS 7 days)	*F*	*P*-value
A	28.20 ± 1.13	26.74 ± 2.06	26.66 ± 2.04		
B	27.80 ± 0.90	26.67 ± 1.84	26.84 ± 1.72		
C	28.13 ± 0.79	25.33 ± 2.55	25.94 ± 2.26		
				3.021	0.052

Data are presented as a number or means ± SD. Multivariate analysis of variance showed that there was no difference in MMSE scores in three groups.

**TABLE 3:** Postoperative incidence of POCD in three groups (%).

Time	Group	Number of cases of POCD	The incidence of POCD(%),95%CI RE (%)	*P*-value
	A	11 (50)	22.0 (12.75, 35.24)	0.031*
3rd day after surgery	B	9 (49)	18.4 (9.98, 31.36)	0.015*
	C	21 (48)	43.7 (30.7, 57.7)	
	A	13 (50)	26.0 (15.87, 39.55)	0.09
7th day after surgery	B	8 (49)	16.3 (8.52, 29.04)	0.004*
	C	21 (48)	43.7 (30.7, 57.72)	

Data are presented as a number, *P < 0.05 vs. group C; *P < 0.05 vs. group C.

### IL-6, TNF-α, NSE, and S100β levels

**FIGURE 4:**
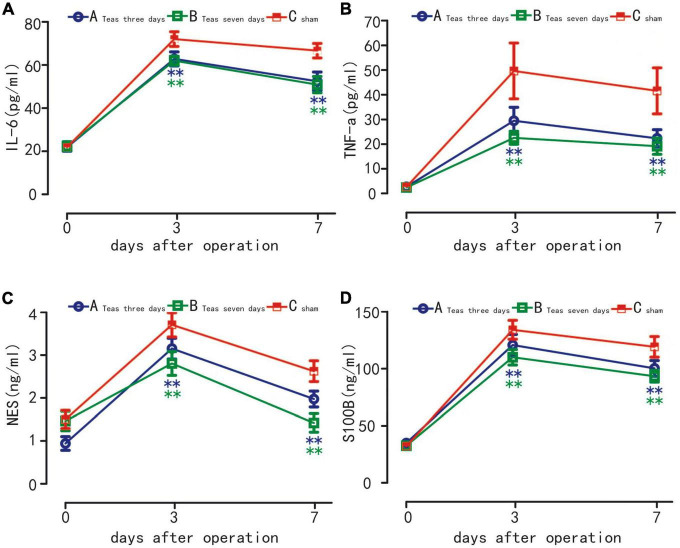
Plasma levels of IL-6 **(A)** TNF-α **(B)** S100β **(C)** and NSE **(D)** in three groups. ^**^P < 0.01 vs. group A at the same time point; ^**^P < 0.01 vs. group B at the same time point.

Compared with T0, the serum levels of IL-6, TNF-α, NSE and S100β were significantly increased in the three groups on postoperative days 3 and 7 (*P* < 0.01). On the 3rd and 7th days, compared with the C group, the serum levels of IL-6, TNF-α, NSE and S100β in the A and B groups were significantly decreased, and the difference was statistically significant (*P* < 0.01). There was no significant difference in the serum levels of IL-6, TNF-α, NSE and S100β between A and B groups (*P* > 0.05) (Figure 4).

## Discussion

Advanced age is an independent risk factor for patients at high risk of POCD (Moller et al., 1998), and the probability of POCD after major surgery in patients older than 60 years is approximately 41.4% (Monk et al., 2008). Lumbar spine disease is a common orthopedic disease in elderly individuals. In the United States, approximately 47.2% of patients aged 60–69 years have lumbar spinal stenosis, and the prevalence gradually increases with age (Epstein, 2017). Massive bleeding, a strong stress response and an inflammatory response are the main complications of lumbar spine surgery, and these may be the root cause of POCD, so it is necessary to intervene in this type of surgery in advance to minimize the occurrence of such complications. Although it is still uncertain whether the inflammatory response is the underlying cause of POCD, the levels of inflammatory factors in peripheral blood (Zhang et al., 2022), the integrity of the BBB (Cao et al., 2018), and the activation of astrocytes and microglia (Li et al., 2020) appear to play an important role in the pathogenesis of POCD.

One of the keys to acupoint electrical stimulation intervention is acupoint selection. Acupoint compatibility refers to the application of two or more acupoints to obtain a more complete therapeutic effect. In long-term clinical practice, similar physicians have summed up a variety of methods, such as the selection of acupoints based on syndrome differentiation, the compatibility of main and auxiliary acupoints, the compatibility of parts, the compatibility of meridians and collaterals, and the compatibility of specific points. Combinations of different acupoints may trigger synergistic or antagonistic effects, thereby inhibiting the effectiveness of treatment (Chen et al., 2012). Cognitive impairment is recorded as “forgetfulness” and “dementia” in ancient literature. The tenet of traditional Chinese medicine is that the brain is the home of the primordial spirit. When the mind is out of use and the brain is damaged, it will lead to cognitive impairment. In the selection of acupoints for the treatment of mild cognitive impairment, most points are on the head, face and neck of the “Du meridian.” Baihui (GV 20) is the meeting point of the bladder, Sanjiao, gallbladder, and liver meridians. Dazhui (GV 14) is the meeting point of the three yang meridians of the hand and foot. The “Baihui” and “Dazhui” points are the “Du meridian.” Studies showed that electrical stimulation of the “Baihui” acupoint can reduce the expression of matrix metalloproteinase-9 (MMP9), the permeability of the BBB, and the occurrence of cerebral edema (Dong et al., 2009; Zou et al., 2015). Electrical stimulation of two acupoints, “Baihui” and “Dazhui,” can reduce the levels of central IL-1β and TNF-α and inhibit the activation of microglia to the M1 phenotype (Han et al., 2017; Liu et al., 2020). In addition to reducing the central inflammatory response, stimulating these two acupoints can also reduce the central oxidative stress response (Shen et al., 2016) and the level of central acetylcholine (Sun et al., 2012) to protect against impairment of cognition. This study showed that electrical stimulation of “Baihui” and “Dazhui” acupoints can reduce the levels of the inflammatory factors IL-6 and TNF-α in the blood of patients and the levels of the neurological damage markers NSE and S100β, indicating that during the intervention process, inflammation is reduced and thus has neuroprotective effects.

There is currently no uniform standard for the duration of intervention with TEAS for reducing POCD. Whether different electrical stimulation durations have different effects on improving POCD is still uncertain. Some studies showed that continuous stimulation from 30 min before surgery to the end of surgery can improve postoperative POCD in elderly patients and play a protective role in neurological function (Liu et al., 2021; Xi et al., 2021; Wei et al., 2022). Generally, TEAS intervention is used in the perioperative period, starting more than 30 min before the operation, to reduce the patient’s pain response when the surgeon cuts the skin. We call this period the “induction period.” However, some researchers believe that if the stimulation time is too long, its therapeutic effect will gradually weaken, that is, there may be a “tolerance effect.” The reason for tolerance may be that prolonged stimulation causes the sustained release of certain neurotransmitters, which act on specific receptors for a long time, resulting in a gradual decrease in the number of receptors or activation of the body’s negative feedback mechanism (Han, 2011). Since the surgery time in this study was approximately 2 h, the intervention time selected in this study was 30 min before the operation and 30 min every day after the surgery. We found that the incidence of POCD was significantly lower in the TEAS group than in the sham TEAS group. There was no difference in the incidence of POCD between 3 and 7 days of intervention, indicating that the prolonged intervention did not reduce the incidence of POCD on an underlying basis, and the duration of postoperative intervention could be selected within 3 days.

The MMSE is a test widely used to assess the severity of cognitive impairment and monitor the progress of cognitive impairment. It is affected by many demographic factors, including age, education level and cultural background (Anderson, 2019; Cardoso et al., 2022), and education level becomes the most influential factor (Wu et al., 2021). In this study, patients with less than 9 years of education were excluded from participating in the MMSE to ensure that they were mostly unaffected by this factor in the study, and the MMSE score of the healthy population was used as the baseline study value to calculate the “Z” score to improve the accuracy of the POCD assessment.

This study has certain limitations. Since it is a single-center study, if further verification of the accuracy of the experiment is needed, then the sample size needs to be increased. Only the impact of TEAS on lumbar spine surgery in the elderly was studied, and the selection of surgery was too singular. In addition, the MMSE scoring method adopted in this study has certain defects, especially the repeated use in a short period of time, which will produce learning effects and have ceiling effects. In future studies, more comprehensive assessment methods should be added to avoid such defects. Furthermore, the observation time of POCD in this study was limited to 7 days after the surgery, and the occurrence and duration of POCD may be longer. The failure to follow up after 1 month and 1 year is also a limitation of this study.

Through the clinical work, we found that, in the orthopedic surgery, especially in the lumbar spine surgery, the probability of older patients with postoperative POD or POCD is very high, and we are always looking for a simple and effective method to reduce perioperative neurocognitive disorders. The equipment required for transcutaneous acupoint electrical stimulation (TAES) is simple to operate. TAES also causes less damage to the patient and less complications. “Baihui” and “Dazhui” acupoints are on the head and back, and the acupoints can be found with special signs. The searching of these acupoints is very simple, so the operation process takes short time so that the patients are more comfortable and more receptive to it. Most importantly, our study found that it was effective in reducing the incidence of POCD and worthy of clinical promotion.

## Conclusion

In conclusion, based on our experimental results, we concluded that TEAS could reduce the level of inflammatory factors in the blood of older patients undergoing lumbar spine surgery and reduce the incidence of POCD, and the intervention time can be more than 3 days.

## Data availability statement

The raw data supporting the conclusions of this article will be made available by the authors, without undue reservation.

## Ethics statement

The studies involving human participants were reviewed and approved by the Ethics Committee of the First Affiliated Hospital of Gannan Medical College (LLSC-2020070203). The patients/participants provided their written informed consent to participate in this study.

## Author contributions

L-FW contributed to the conception of the manuscript, acquisition and analysis of data, drafting, editing and critically revising the manuscript and performed the literature search, final approval of the work, and agreed to be accountable for all aspects of the work. J-MY contributed to the design of manuscript. LC and M-LZ critically revised the pathology report and edited the manuscript, final approval of the work, agreed to be accountable for the work, and contributed to the design of manuscript. W-DL, B-YW, M-LG, and J-SZ revised and edited the manuscript and assisted in literature review, final approval of the work, and agreed to be accountable for the work. All authors read and approved the final manuscript.
